# A Review on Mitigating Fear and Aggression in Dogs and Cats in a Veterinary Setting

**DOI:** 10.3390/ani11010158

**Published:** 2021-01-12

**Authors:** Stefanie Riemer, Carmen Heritier, Ines Windschnurer, Lydia Pratsch, Christine Arhant, Nadja Affenzeller

**Affiliations:** 1 Companion Animal Behaviour Group, Division of Animal Welfare, Vetsuisse Faculty, University of Bern, 3012 Bern, Switzerland; 2 Independent Researcher, 88045 Friedrichshafen, Germany; carmen.heritier@outlook.de; 3 Institute of Animal Welfare Science, University of Veterinary Medicine Vienna (Vetmeduni Vienna), 1210 Vienna, Austria; ines.windschnurer@vetmeduni.ac.at (I.W.); christine.arhant@vetmeduni.ac.at (C.A.); 4 Veterinary Behaviour Consultant, 1200 Vienna, Austria; office@tierverhaltenspraxis.at; 5 Department of Companion Animals, Clinical Unit of Internal Medicine Small Animals, University of Veterinary Medicine Vienna (Vetmeduni Vienna), 1210 Vienna, Austria; nadja.affenzeller@vetmeduni.ac.at

**Keywords:** stress, fear, anxiety, aggression, veterinary visit, low-stress handling, counterconditioning, behaviour modification, anxiolytic medication, psychoactive drugs, dogs, cats

## Abstract

**Simple Summary:**

The majority of dogs and cats are fearful during veterinary visits, and some individuals may show aggression as a result. We review ways to avoid negative experiences and promote positive emotions in animals visiting the veterinarian. Whenever an animal is in the practice, the veterinary team should endeavour to make the visit as pleasant as possible, by using non-threatening body language and by creating positive associations. High-value food (unless an animal needs to be fasted) or toys should be used generously throughout the visit. In the interaction with the animals, low-stress handling methods, brief pauses and adjusting the procedure based on the animal’s body language help them to feel secure. Distractions can be used to minimise perceived pain such as from injections. If a known painful area needs to be treated, pain killers are advised. For animals that are very fearful, several medication options are available that can be given prior to the veterinary visit to help them with their fears. With reward-based training, animals can learn to accept veterinary procedures. A stress-free veterinary visit benefits all involved parties—the animals, their owners, as well as the veterinary team.

**Abstract:**

A high proportion of dogs and cats are fearful during veterinary visits, which in some cases may escalate into aggression. Here, we discuss factors that contribute to negative emotions in a veterinary setting and how these can be addressed. We briefly summarise the available evidence for the interventions discussed. The set-up of the waiting area (e.g., spatial dividers; elevated places for cat carriers), tailoring the examination and the treatment to the individual, considerate handling (minimal restraint when possible, avoiding leaning over or cornering animals) and offering high-value food or toys throughout the visit can promote security and, ideally, positive associations. Desensitisation and counterconditioning are highly recommended, both to prevent and address existing negative emotions. Short-term pain from injections can be minimised by using tactile and cognitive distractions and topical analgesics, which are also indicated for painful procedures such as ear cleanings. Recommendations for handling fearful animals to minimise aggressive responses are discussed. However, anxiolytics or sedation should be used whenever there is a risk of traumatising an animal or for safety reasons. Stress-reducing measures can decrease fear and stress in patients and consequently their owners, thus strengthening the relationship with the clients as well as increasing the professional satisfaction of veterinary staff.

## 1. Introduction

Veterinary care is an essential element of maintaining good welfare of companion animals. However, studies indicate that a great majority of dogs and cats show signs of fear when visiting the veterinarian [1,2,3], making veterinary visits stressful also for their owners [4]. Indeed, 28% of cat owners and 22% of dog owners reported that they would consult the veterinarian more often if the visit was not associated with so much stress for their pet [4]. Animals’ stress associated with the veterinary visit can distort physiological measurements, hamper the physical examination and, in case of aggression, pose a risk to the veterinary team [5]. Veterinarians are thus faced with the challenge of carrying out important medical procedures, some of which may be painful, while also considering the emotional well-being of their patients. Although a classic paper investigated fear-related behaviour in dogs at a veterinary clinic already in 1981 [6], awareness on how individual animals experience veterinary visits and how negative experiences can be counteracted has been increasing only relatively recently [7,8]. Here, we provide a comprehensive review of causes for and measures to reduce, or ideally prevent, fear and subsequent aggressive behaviour in dogs and cats at the veterinarian, based on controlled studies and—in the absence of such studies—peer-reviewed guidelines or expert texts.

### 1.1. Fear, Anxiety and Stress—Evolutionarily Adaptive

From a biological point of view, fear, anxiety and stress reactions are highly adaptive, as they organise behavioural responses to avoid or cope with threat [9,10]. In dangerous situations, two major behavioural patterns can be distinguished. The “fight-or-flight response” constitutes an active coping strategy, while “freezing” is a passive coping strategy [11]. Which behavioural reaction the animal chooses when it feels threatened depends on the one hand on its personality [12], and on the other hand, on the situation, for example the distance to the alleged threat, the availability of an escape route and the perceived degree of danger [11,13].

Both fear and anxiety are unpleasant emotional reactions to the presence (fear) or potential presence (anxiety) of a threat [14]; however, there is no empirical data on distinguishing expressions of anxiety vs. fear in dogs and cats (c.f. [15,16]). Therefore, and since anxiety promotes fear and vice versa [13,17], we subsequently use the term “fear” to denote both fear and anxiety responses.

The term stress refers to the organism’s response to a stimulus or event, often referred to as “stressor”, that poses a potential threat and triggers a physiological and behavioural response, including activation of the sympathetic adrenal medullary axis and the hypothalamic-pituitary-adrenal cortex axis [18,19]. For example, in cats, the transport to the veterinary practice and routine visits for vaccinations can elicit significant physiological changes, including elevations in blood pressure, rectal body temperature, pulse, respiratory rate and blood glucose, as well as a change in the cortisol-creatinine ratio [5,20,21,22]. In some healthy dogs, the urinary corticoid:creatinine ratio increased following a veterinary visit to a level that would be consistent with hyperadrenocorticism [23]. A reduced effectiveness of sedation [24] and an increased risk of anaesthetic complications [25] are to be expected in stressed animals.

### 1.2. Stressors in a Veterinary Setting and Individual Responses

Potential stressors during a veterinary visit include exposure to other animals and unfamiliar people [26,27], odours (e.g., disinfectants and alarm pheromones from other animals), noises (barking dogs, clippers, alarms etc.), and smooth or slippery surfaces such as floor coverings or metal examination tables [27]. For cats, leaving the familiar environment is often particularly stressful [28,29]. The unpredictability of the situation and the loss of control during restraint constitute a major source of stress [29,30]. In addition to an immediate reduction in welfare, it should be considered that every negative experience may increase fear during subsequent veterinary visits [2,3]. Moreover, the experience of discomfort or pain, especially in response to physical manipulation, might lead to escalated behaviour such as aggression [31,32,33,34,35].

To reiterate, aggressive (“fight”) responses are an inherent component of the biological fear reaction [11], and in a veterinary context, aggression ensues most frequently due to fear, pain or fear of pain [32,36]. As long as it is possible to avoid a perceived threat, many animals may initially freeze or hide. However, when the danger is perceived to be too close, the animal may attempt to flee, and if there is no escape route, it may try to attack [11,13]. However, freezing may also again be shown when the animal realises that no escape is possible [11].

Since aggression is one of the indicators that an individual’s fear threshold was exceeded, interventions to reduce fear in animals usually apply in the same way to animals showing aggressive responses, and veterinary staff should be no less compassionate towards animals reacting aggressively than to those that are overtly fearful. Thus, by use of low-stress handling methods, adjusting to already subtle indicators of distress, creating positive associations, and where necessary use of anxiolytic medication, the animals’ emotional state can be improved so that heavy restraint becomes unnecessary, the animals feel less cornered, and the risk of aggression, and thus potential injury to veterinary staff, is reduced.

### 1.3. Identifying Stress and Fear in Dogs and Cats

The recognition of (subtle) stress signs enables the veterinary team to respond early to the first signs of discomfort and to adjust the interaction accordingly. Such signs include the shifting of body weight away from the trigger, crouching, and increased muscle tension on the face and throughout the body [27,29,37,38]. Gaze avoidance, changes in activity level (freezing or agitation), distance-increasing attempts and whether an animal is willing to consume offered food give further indications about the patient’s stress level [27,29,37,38].

Fearful dogs usually have their ears directed backwards or flattened. Further signs of fear in dogs include a lowered posture, a lowered or even tucked tail, panting, increased salivation, trembling and emptying of the anal glands [3,15,37,39]. Lip licking is a commonly used appeasement signal in a social context [15,37]. However, when feeling severely threatened, dogs may freeze completely [38] and no longer show other communicative signals [37]. Defensive aggression, such as stiffening, staring, growling, and snapping or biting, may ensue [38].

Fearful cats typically make themselves smaller. The ears are directed backwards, sideways or flattened, and the pupils are enlarged [29]. When lying or sitting, the tail is kept close to the body. Tail swaying in cats indicates agitation, annoyance or high arousal; often high vigilance can be observed from ear movements [39]. When displaying defensive behaviour, cats may crouch, hold the ears downwards or sideways and hiss, growl or shriek, but they can also stand on tiptoes, holding the tail stiffly upwards or downwards, and appearing larger due to piloerection [39,40]. Aggression is often imminent when the ears are swivelled sideways so that the inner pinnae are displayed and the pupil is oblong. Moreover, note that purring in cats is not only shown due to contentment, but also when sick [39].

While freezing and fleeing are usually the primary coping strategies when fearful, when no escape is possible in a hospital setting, the animal may feel increasingly threatened and eventually may resort to aggression [39]. Dogs may growl, lunge and bite. Cats may hiss, growl, swat with their paws and bite [36]. Notably, signs of fear, such as backwards-directed ears, may disappear or only be shown during earlier stages as the arousal heightens and the aggressive behaviour intensifies [36]. There may also be a learned component to aggressive behaviour at the veterinarian’s: if this strategy was successful in stopping an unpleasant situation during a previous visit, the animal may opt for the “fight” strategy again the next time [36]. Needless to say, such escalation should be prevented whenever possible, by considerate handling (see below Section 3) and adjustments according to the animal’s more subtle signs of distress.

## 2. Creating a Low-Stress Environment

### 2.1. Reception Area and Waiting Room

**Figure 1:**
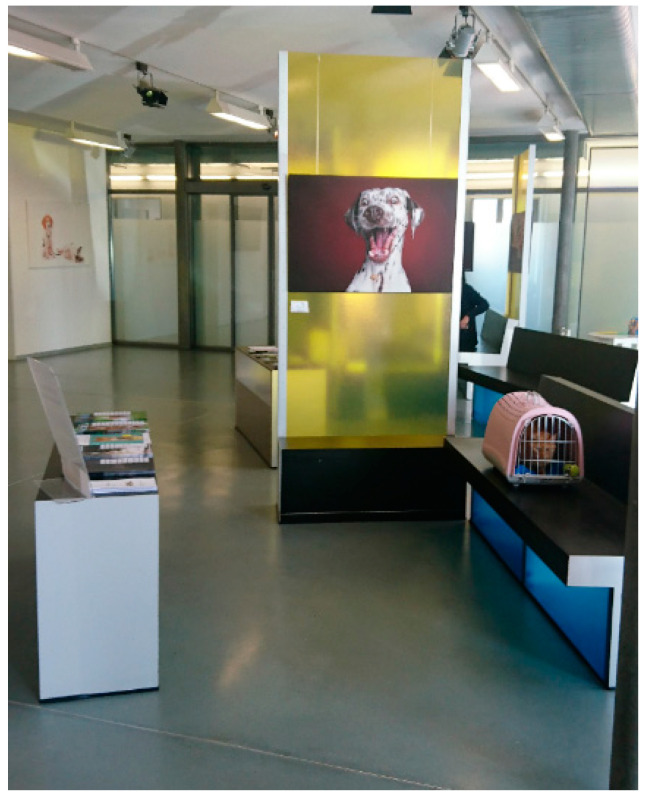
A spacious waiting area with visual barriers is ideal. Due to the layout of the benches, waiting animals do not need to face each other. Cat carriers should be placed on an elevated surface; ideal would be an additional covering of the carrier. Photo: Stefanie Riemer.

While the examination and treatment represent the most stressful parts of a veterinary visit [3,41], a high percentage of dogs and cats are already fearful in the waiting room [1,2]. Even completely harmless activities, such as weighing on the scale, were found to increase signs of stress in 53% of dogs [38]. Many dogs and cats are stressed by the presence of conspecifics or other species both inside and outside of the practice [42,43]. These difficulties are exacerbated if it is not possible to avoid other animals in confined spaces. Thus, the waiting area should be set up to allow maximum distance between animals and should include barriers to reduce visual contact between patients [7] (Figure 1). In the waiting room, like throughout the clinic, sound-absorbing tiles, rubberised floors or wall panels and solid doors are recommended to absorb potentially aversive sounds (reviewed in [8]).

Whenever possible, separate premises are recommended for dogs and cats, as the presence of dogs can cause fear in cats [27,36,42]. If this is not possible due to the local infrastructure, visual barriers can be used to section off ‘cat zones’ [44]. Another option to avoid exposure to dogs is to offer consultation hours for cats only [29]. Elevated spots for cat carriers, for example extra chairs or shelves, and covering the carrier with a cloth, can reduce cats’ stress in the waiting area [29,44].

If a dog is known to have problems with other dogs, this should likewise be taken into account when making appointments, so that the dog can be booked in at off-peak times. In order to minimise time in the waiting room, owners can be advised to wait in the car or to take the dog for a walk when a longer waiting time is foreseeable [32]. Waiting in other areas has also been demonstrated to be effective in alleviating stress: after waiting in a garden outside the clinic with their owners for twenty minutes, dogs had lower heart rates and cortisol levels than dogs that spent the same time in the waiting room [45]. Fewer stress signs also occurred when waiting in an empty examination room as compared to the waiting room [46].

**Figure 2:**
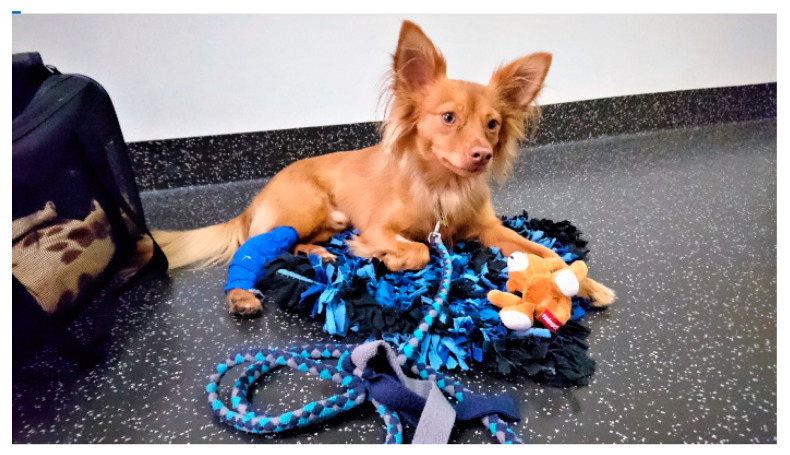
Owners can be advised to bring a blanket from home and to distract the animal with food or toys in order to decrease stress during the waiting time. However, the potential for resource guarding when using toys or chews needs to be considered. Not suitable when sufficient spacing between animals in the waiting area is not possible. Photo: Stefanie Riemer.

Fear prevention can commence by reception staff offering treats to the animal upon entering the practice (taking into account individual dietary requirements) [36]. Weighing, a source of stress for approximately every second dog [47], is more comfortable for dogs if the scale has a non-slip surface and is not set up in a corner [27,40]. Fear of weighing can be reduced by encouraging and rewarding dogs for stepping on the scale on their own [40]. Easy-to-clean dog beds that insulate from the cold floor can improve dogs’ comfort [48], even more so if owners bring their own blanket or dog bed [29,36]. Owners can furthermore bring chews or toys to occupy the dog during the waiting time [27,36] (Figure 2).

### 2.2. Examination Room

More than three-quarters of dogs observed in a veterinary practice were categorised as fearful in the examination room [3], with the highest occurrence of fearful behaviour on the examination table. This can be attributed to dogs not being used to standing on a table, but in particular to negative associations, which can quickly form with stimuli that were present during a previous negative experience, such as a painful treatment. Such negative associations can also include the veterinary staff or the treatment room (reviewed by Döring et al. [3]). Counterconditioning can be used to change such negative associations and replace them with positive ones [3] (see below Section 9.1).

**Figure 3:**
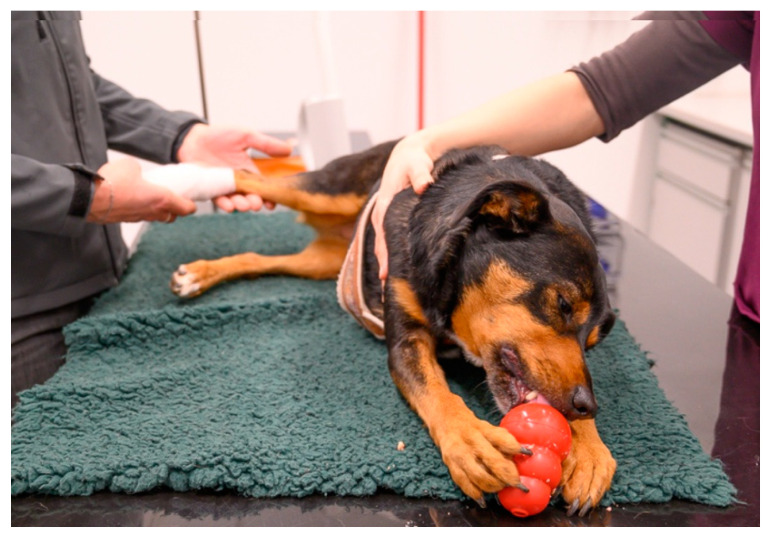
A non-slip mat improves the animal’s comfort. By feeding the animal during the examination or treatment, positive associations can be created. Photo: Christine Arhant, Vetmeduni Vienna.

**Figure 4:**
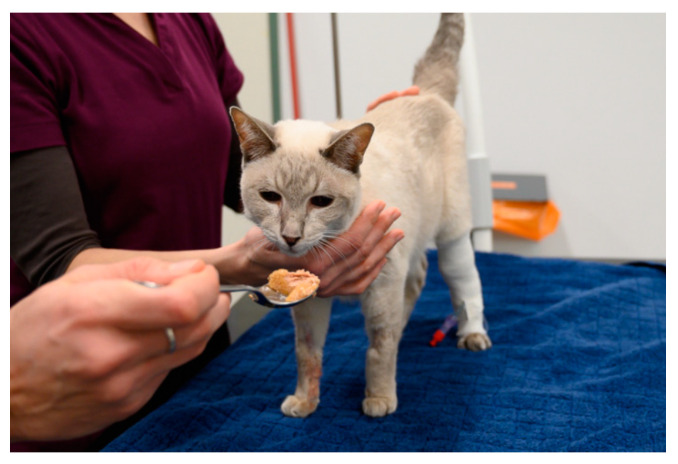
Use of high-value food (or toys) can increase cooperativeness and promotes positive emotions. Photo: Christine Arhant, Vetmeduni Vienna.

**Figure 5:**
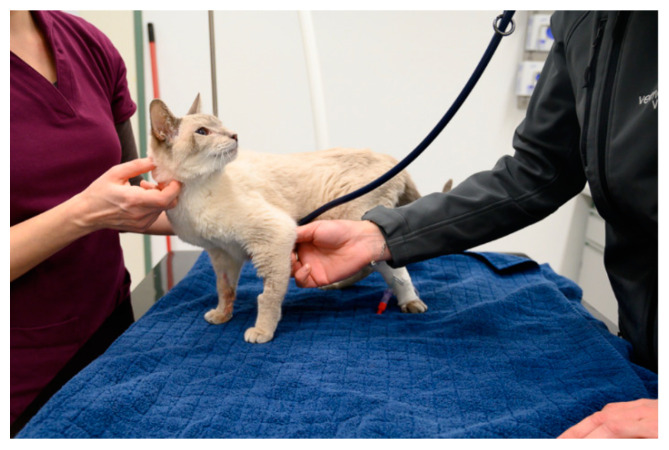
Performing the examination in a position that is most comfortable for the individual and using the minimal amount of fixation needed improves the patients’ compliance and well-being. Photo: Christine Arhant, Vetmeduni Vienna.

The examination/treatment should take place where the animal feels most comfortable [29,49]. Many dogs are more relaxed when examined on the floor than on the table [3]. Cats often stay calmer if they can remain in the bottom of the carrier during the examination; therefore, carriers with a removable top half are ideal [29,36,49,50,51,52]. Slippery, hard and cold surfaces can cause discomfort, which can be avoided by the use of non-slip mats or soft foam pads or by the provision of towels and blankets (using a new, clean one for each patient) [24,53] (Figure 3, Figure 4 and Figure 5). Owners can also be encouraged to bring their own blankets or towels, as the familiar smell is comforting [28].

Cats and small dogs can also be examined on the veterinarian’s or the owner’s lap [29,36,37]. For some individuals with severe negative associations with the examination room, performing the examination outside may be the least stressful option [24].

‘Taking the animal to the back’ for minor procedures should be avoided. Moreover, [54] performed a crossover trial in which dogs were examined in an exam room in the presence of their owners vs in the owner’s absence in a common treatment area. The common treatment area included two exam tables, two tables for dental procedures, cages holding cats and small dogs as well as a row of large dog runs. Compared to the examination in the exam room, dogs exhibited a significantly elevated behavioural score for measuring fear, anxiety and stress, and heart rate was on average 20 bpm higher in the common area [54].

Similarly, a study using the same methodology with client-owned cats demonstrated that heart rate was 30 bpm higher in the common treatment area than in the exam room with the owner. Additionally the behavioural score during the examination was higher than baseline in the common area, whereas no significant change was noted in the exam room [55].

These studies demonstrated that, whenever possible, examinations or minor procedures should be performed in the exam room, with the owner present [54,55]. If, for any reason, the owner’s presence is not desirable; it is recommendable to request the owner to leave the examination room and perform the intervention in that room, instead of taking the animal to the common treatment area [55].

### 2.3. Sensory Considerations

When stressed, dogs and cats can produce alarm pheromones, such as from their paw pads and, in dogs, anal gland secretions, which could subsequently have an alarming effect on other patients [26,56]. Thus, surfaces must be cleaned not only for hygienic reasons, but also in order to minimise odours from other animals [24], using cleaning agents that lead to the denaturation of the corresponding components [26]. Since the smell of disinfectants can be perceived as aversive by animals [27], sufficient time should be allowed for cleaning agents to evaporate [24]. Good ventilation of the rooms can also reduce aversive odours [27].

Although the examination and handling generally constitute greater stressors for animals than the ambient noise [41], it is advisable to keep the noise level as low as possible. This can be accomplished by structural measures, e.g., use of sound-absorbing materials on walls, floors and doors, as well as by speaking with a quiet and calm voice and avoiding unnecessary ambient noise, such as from slamming cage doors [24,57]. In addition, playing soothing background music (classical music or commercially available recordings) has been recommended [24]. One study indicated that music specifically designed for cats, but not classical music, was associated with fewer stress indicators and reduced resistance to handling in cats during a clinical examination compared to no background music [58]. In dogs, some studies suggest a possible calming effect of music (reviewed in [59]), but a study performed in a veterinary context could not demonstrate a reduction in fearful or aggressive behaviour [46]. However, the owners found the waiting time more pleasant, and the veterinarians indicated that they enjoyed the music [46]. Further studies are needed to address species-specific preferences, as well as individual responses [59].

## 3. Low-Stress Handling and Creating Positive Associations

### 3.1. Treatment Plan

A treatment plan should be made for each individual patient, taking into account their behavioural evaluation and the planned procedures. On this basis, decisions should be made regarding the location of the examination (e.g., table vs. floor), the degree of restraint, the use of food and toys, the use of aids and the assessment of a potential safety risk [24].

The planned examinations and interventions should be ranked according to invasiveness, and the examination should begin with the least unpleasant step, with the most unpleasant procedure performed last [29]. If an animal shows severe distress and treatment is not urgent, it should be considered whether interrupting the procedure and setting up a new appointment would be more conducive to the psychological health of the animal (and its owner). The animal could then be prepared for this next visit either by pre-visit anxiolytic medication or by cooperative care training in which the animal is trained through positive reinforcement to accept handling and treatment [24,60]. The use of sedation is preferable to creating a traumatic experience. Since sedation is less effective when animals are in an elevated state of arousal, it should be administered before fear escalates, if foreseeable [24,38].

Considerations that are most important when animals are known to be fearful or to react aggressively include preparing all the equipment needed before attempting to handle the patient and being aware of possible ‘problem zones’ when handling the animal, since pain or the expectation of pain increase the likelihood of aggression [61].

### 3.2. First Contact with the Animals

When greeting dogs, it would be ideal if the dog initiates the first contact on its own initiative [62]. Contrary to the common recommendation, it is not advisable to stretch out the hand to make contact or to hold it in front of the dog’s nose to sniff [49]. Instead, it is less threatening for the animals if the hands are held loosely on the side of the body [62]. Many gestures performed by humans with friendly intentions can be perceived as threatening by animals, such as direct eye contact, a frontal approach, and leaning over the animal [24,49]. Therefore, turning sideways when greeting an animal, or approaching from the side if no approach is initiated by the animal, is recommended. Nervous dogs can be greeted sitting or squatting, with the body weight shifted away from the animal. For safety reasons, the face should be kept out of reach of the animal’s face [7,24,42]. Using the animal’s name and talking to it in a friendly voice can promote familiarity and security [61]. A positive bias is facilitated by the use of treats [62]. With confident dogs, treats can be handed directly; with shy animals, they should be offered or tossed on the floor to avoid an approach/avoidance conflict [48]. It is best to commence the examination where the animal is most likely to accept touch [24].

Cats should never be dragged or even shaken out of their carriers, an extremely frightening experience due to the complete loss of control [29,37,52]. A little patience often pays off: in one study [52], more than half of the cats left the carrier within three minutes. When the cat has exited its carrier, it is beneficial to allow it to explore the treatment room while the medical history is taken (provided there are no inaccessible hiding places or escape possibilities) [29]. The lower part of the carrier should remain accessible throughout the examination. More than 90% of the cats like to use it as a retreat during the clinical examination [52].

In carriers with a removable top, the cat can remain in the lower part during the entire examination [49,50,51,52]. A towel placed over the lower part and over the cat serves as a hiding place and may promote a sense of security [49,51]. If examining in the carrier is not an option and the cat shows no intention to exit the carrier after a few minutes, luring it with high quality food or toys can be attempted [36]. Should it be necessary to manually remove the cat from the carrier, reaching around the caudal abdomen and hind legs and applying light pressure can motivate calm cats to move forward [29]. For timid animals, the use of a towel may be helpful to gently pull them forward [49].

### 3.3. Considerate Body Language

Smooth, slow handler movements and calm speech are conducive to the patients’ relaxation [24,40], while sudden position changes can be perceived as startling or threatening [62]. During contact, repeated letting go and touching of the animal should be avoided. Instead, the hands can be slid along the body to the area of interest [24]. Although physical contact and petting can have a calming effect on animals [63], this should only be done by the veterinary team if the animal obviously enjoys it [7]. Periodic brief pauses of the examination and enabling some play or pleasant activity can lower arousal and promote more positive emotions [36].

### 3.4. Creating Positive Associations

Whenever an animal is at the veterinary practice, it should be endeavoured to create positive associations, which can often be achieved easily by the use of food or toys—these should not be limited to the end of the visit, but can already be used generously during the examination or treatment, both to distract the animal and to elicit positive emotions [53,60,62] (Figure 3 and Figure 4). For example, animals can lick delicious paste while on the table and receiving an injection, or the owner can offer a handful of treats in quick succession [36].

### 3.5. Balancing Physical and Emotional Health

The emotional state of the patients should be continually evaluated. For example, Herron and Shreyer [24] propose a colour scale system for this purpose: As long as the animal is within the green zone, it is relaxed, able to take food and safe to handle. Yellow indicates that the patient is anxious and alert. While the animal is more likely to retreat at this stage, aggressive reactions cannot be excluded if it is provoked further [24]. Here, steps should be taken to alleviate the animal’s stress, e.g., pausing the examination, adjusting the restraint technique or location of the animal, involving the owner, and providing distraction with incentives such as food or toys [24,60].

A red rating refers to an animal that appears to fear for its life and is ready to attack. Safe handling is not possible under these circumstances. If it is essential to proceed the examination and/or treatment for health reasons, chemical restraint should be performed prior to any continuation of the procedure, both to avoid traumatising the animal and to minimise risk to the staff [24,60]. Note, in this context, that sedation is less effective when the animal is already agitated; therefore, if needed, sedatives should ideally be administered prior to any escalation [24,38]. For non-essential visits and when owner compliance can be expected, rescheduling the visit and preparing the animal with a handling plan and/or pre-visit anxiolytic medication is recommended [24,60].

## 4. Restraint Methods

The more comfortable an animal feels in its environment and during manipulation, the more likely it is to remain calm and cooperative [49], which not only improves the individual’s welfare, but also reduces risk to the staff, since forceful handling is one of the contexts associated with an increased likelihood of aggression [3,32,36]. The examination or treatment should therefore be carried out in a position and using a restraint method which causes the least stress to the animal, in order to perform the respective intervention lege artis, while ensuring the safety of all involved [49] (Figure 5). The use of food enhances the animal’s cooperativeness so that minimal restraint is often sufficient [40] (Figure 3 and Figure 4).

Unpredictability and loss of control are among the strongest psychological stressors [64]. Accordingly, full body restraint (e.g., lateral recumbency with the fore and hind legs held firmly) or scruffing the neck lead to stronger defensive reactions and more escape attempts and signs of stress than minimal fixation, in which the animal is allowed to remain in any body position and is only gently prevented from moving away from the examiner [29,30,37] (Figure 5). Alternative methods of head control, such as the use of towels or blankets, should be preferred [29,49] (see below Section 4.1.1).

If an animal resists restraint for more than two to three seconds, interrupting and repositioning may be helpful. If the animal remains uncooperative after two to three further attempts, a different handling approach should be attempted [49]. For urgent interventions, sedation should be performed before the situation escalates [29]. If the procedure is simply continued by using force, this carries a high risk of increasing fear and aggression during the next visit, as well as the potential for staff injury [3,32,36]. Reactions such as a strict “no”, punitive measures or forceful restraint further increase the risk of aggressive behaviour [36,65]. Thus, for both animal welfare and staff safety, stress-reducing measures should constitute an essential part of everyday veterinary practice, and if these are not sufficient to allow a safe examination, anxiolytics and chemical restraint should be considered whenever a situation may entail excessive fear or the display of aggressive behaviour [36].

### 4.1. Tools to Facilitate Restraint and Safety

#### 4.1.1. Towels, Blankets, and Alternatives

**Figure 6:**
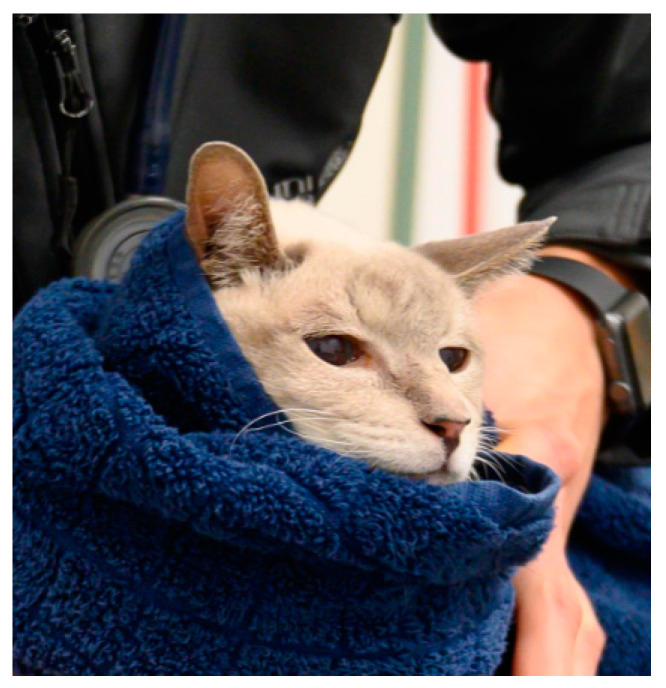
Towels and blankets can be used in various ways for safe restraint of cats and small dogs. Photo: Christine Arhant, Vetmeduni Vienna.

Towels and blankets are often recommended for the restraint of cats and smaller dogs, to shield animals from visual stimuli and to create a sense of hiding [36,37,54] (Figure 6). Providing a protective barrier between person and patient, towel wraps can constitute effective safety measures [36], as they can also protect against cats‘ claws. They can furthermore be used to control the head, especially in small and brachycephalic dogs and in dogs that show fear of the muzzle [24].

‘Cat bags’ can be used as a means to restrain cats in similar ways as with towels, but some concerns have been raised that placing the cat into the bag may be difficult and that fitting it too tight might be distressing, whereas restraint might not be sufficient with a loose-fitting bag [29]. Thus, using towel techniques was suggested to be preferable for this purpose [29].

Anxious and agitated animals can be calmed by placing a towel over their head, suppressing visual stimuli [36]. Another way to achieve the latter may be the use of ‘calming caps’, which are placed over dogs’ heads, including the eyes, to reduce visual stimulation [24,40]. One potential advantage is the prevention of anticipatory anxiety, as the dog does not see preparatory actions leading to a procedure. Another use might lie in preventing aggressive dogs from seeing conspecifics or people when kennelled or when moved within the hospital [24]. However, the effectiveness of calming caps in reducing animals’ distress has not yet been researched.

#### 4.1.2. Muzzles and Alternatives

Muzzles constitute important tools to ensure the safety of all involved parties [66], reducing the risk of a bite and thus enabling veterinary staff to be more confident and calmer in the interaction [36]. Muzzles must not be abused to manhandle a resisting dog, and to carry out a procedure despite the dog’s struggling [48]. In such cases, other measures, such as short-term sedation, pre-visit medication, and in the longer term behavioural training must be used [49].

Since stressed dogs often pant [67], muzzles that restrict the dog’s ability to open the mouth and thus panting, such as soft nylon muzzles, are only suitable for very short-term use, such as during an injection. Unsuitable muzzles may restrict normal breathing and thermoregulation, which can, in the worst case, become life-threatening [36]. Only muzzles that do not obstruct air circulation and are deep enough to allow unrestricted opening of the mouth and panting are suitable for prolonged wearing [68].

**Figure 7:**
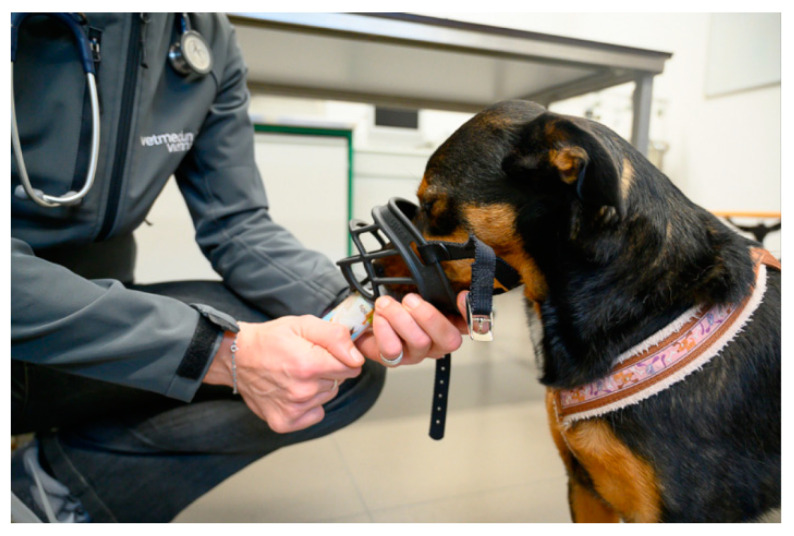
Muzzles increase safety. Food can be used to entice the dog to place its nose into the muzzle and should be offered repeatedly during wearing in order to create positive associations. Photo: Christine Arhant, Vetmeduni Vienna.

Ideally, every dog would have received muzzle training by the owner. For untrained animals that do not need to be fasted, smearing attractive food inside the muzzle is helpful. The muzzle is then offered from below, slipped over the nose and secured as quickly as possible, while the dog licks the food inside the muzzle [24,49]. In order to promote a positive association with the muzzle, food should be offered during the entire duration of the procedure [49], e.g., by feeding paste from a tube or a large syringe [7] (Figure 7).

Cat muzzles cover both mouth and eyes [24]. However, unlike dog muzzles, cat muzzles can hardly be adjusted to the animal, and cats usually do not receive muzzle training. Soft nylon muzzles are a possibility if visual shielding is the primary aim [24,29], whereas hard plastic muzzles are more suitable for the prevention of bites [24,36]. The ball-shaped “Air Muzzle” (Air Muzzle, SmartPractice, Phoenix, AZ, USA), which can be used for small and especially brachycephalic dogs and cats, encloses the entire head and enables normal breathing. Its advantage is that the handler’s hands stay protected behind the device when putting it on [36].

Still, the use of towel wraps may impose less stress on cats or small dogs while achieving good head control [24,36,49]. Another alternative to control the head of dogs and cats and increase safety when handling difficult animals is the use of Elizabethan collars [24,36].

#### 4.1.3. Tools That Should Only Be Used When Alternatives Are Not Feasible

The use of “clipnosis” in cats is under debate [29], with some contradictory results on the welfare implications reported [30,69,70,71,72]. It causes an inhibition of behaviour and therefore immobilisation in cats (although responsivity differs between individuals) [71,72], but it has been controversial whether this behavioural inhibition actually reflects calming or freezing (c.f. [24]). One study suggested that cats showed a lower heart rate and lower pupil dilation when clipnosis was applied compared to scruffing [72], but another study indicated that clipnosis is similarly stressful as full body restraint, the restraint method associated with the most negative reactions, and more stressful than scruffing [70]. In this study, cats showed increased pupil dilation and increased vocalisations at similar levels during full body restraint and clipnosis, whereas scruffed cats vocalised less. Thus, it was concluded that full-body restraint, clips and scruffing are aversive to cats and should be avoided as much as possible, so that manual scruffing might be the method of choice when minimal restraint is not sufficient [70]. Nonetheless, as described above, the use of anxiolytics and chemical restraint might be preferable in such situations [36].

If nets are to be used at all, this should only be the case in special circumstances, and never for longer than a few seconds (e.g., to administer an injection) [29]. With very aggressive or feral cats, it has been suggested that using nets for capturing may incur less stress than forceful manual handling (such as scruffing), while also increasing the handler’s safety [36], but some experts hold the opinion that their use is never justified [29].

Dog catchers (poles with wire loops that can be thrown over the animal’s head and tightened) should only ever be used if there is no other option, for example when there is no other way to remove a highly aggressive dog from the kennel. This is extremely stressful for the animal and carries a risk of injury with improper handling and should therefore be avoided wherever possible [61].

Similarly, the use of crush cages is only intended for emergencies to perform an injection quickly and safely [48,61]. Pressure in the crush cage should only be applied for as long as necessary for the injection [48]. Before and after the injection, both the cage and the net should be covered with a cloth to block visual stimuli [24].

Gauntlets should only be used under special circumstances and in combination with other tools such as nets and towels [49]. The gloves provide protection for arms and hands, but can cause additional stress to the animals, and dexterity is limited during wearing. Thus, they should only be used to restrain patients or to remove them from their carrier or cage if no less stressful alternative is available [48,49].

## 5. Reducing the Perception of Pain

### 5.1. The Power of Distractions

A number of paediatric studies have tested interventions to reduce pain in human infants during injections or vaccinations [73]. Both tactile and cognitive distractions have been shown to be effective in reducing perceived pain [73]. Examples of tactile stimulation include vibration, but also the passive movement of a body part [73,74,75]. The reduction of pain through simultaneous tactile stimulation can be explained by the gate control theory [76]. According to this theory, thin nociceptive nerve fibres (C-type) are inhibited by the simultaneous activation of thick nerve fibres (A-type), which process touch stimuli [77]. Consequently, tactile stimulation slightly preceding and continuing through an injection such as tickling, light tapping or massaging likely contributes to a reduction in pain also in animals [7].

Cognitive distractions, which draw attention to something else, are also effective in human studies to reduce pain perception [73,78]. In pets, this could be achieved by the simultaneous administration of food, in well-trained animals also by performing a trained behaviour, such as resting the chin on the owner’s hand [7]. The use of food can further contribute to a positive association with the situation (counter-conditioning) [49,53]. By using distraction techniques, negative experiences can thus be avoided, and the likelihood of aggressive reactions can be reduced.

### 5.2. Topical Analgesics

The application of EMLA cream (active ingredients lidocaine/prilocaine) can reduce defensive movements in dogs during catheterisation; however, an application time of 60 min was necessary (no observable difference to placebo after 30 min) [79]. In cats, the efficacy of EMLA cream already 30 min after application was demonstrated in cats undergoing jugular blood sampling: the stress score in cats receiving the EMLA cream was significantly lower than in the placebo group, and withdrawal movements were observed in only one of nine cats of the EMLA group, compared to seven of nine in the control group. Moreover, the blinded clinicians rated jugular venipuncture as easy in eight of nine cats having received EMLA and one of nine cats in the placebo group. In already sedated cats, a positive effect of EMLA on struggling during jugular catheterisation has been reported just 20 min after application [80]. For jugular catheterisation in unsedated cats, there was also a tendency for less struggling one hour after EMLA application, but the level of statistical significance was just missed [81]. In addition, it was observed that some cats did not react to the initial insertion of the needle, but to the progression of the catheter into the vein, so that sedation was necessary for successful catheterisation [81]. Thus, the results support the usefulness of EMLA in clinical practice for blood sampling in cats, while success was somewhat lower when catheterisation was necessary without prior sedation. However, if animals subsequently need to undergo anaesthesia anyway, intramuscular sedation as a standard procedure before further invasive procedures such as the insertion of a venous catheter has been recommended in order to minimise the risk of a negative experience [60].

A possible explanation for the longer application period necessary to achieve a sufficient analgesic effect in dogs compared to cats might lie in differences relating to the skin, but also behavioural species differences. Furthermore, stress-induced peripheral vasoconstriction could influence the effectiveness of topical analgesics [82].

When examining an animal that is known to be affected by a painful condition, or when performing procedures associated with pain such as ear cleaning and anal sac expression, pre-emptive analgesia can reduce and ideally prevent pain and thus ameliorate the negative experience and avoid potential aggressive reactions [39].

### 5.3. Optimised Use of Needles

To facilitate blood sampling, using butterfly needles is often recommendable (reviewed in [8]).

For injections, it is advised to use the smallest gauge needle that is practical for the respective purpose, and to use a new needle after drawing the medication in order to ensure that the needle is sharp [8,27].

## 6. Minimising Non-Painful Discomfort

When medication needs to be administered orally, hiding it in palatable treats would be optimal. If this is not possible, a pill administrator or pill gun is useful by avoiding the medication’s contact with the tongue [24].

To prevent animals from scratching or licking wounds, products that minimise discomfort while ensuring safety should be chosen, e.g., protective clothing such as recovery suits or Elizabethan collars made of soft material instead of plastic cones [48,83].

## 7. The Owner’s Influence

Dogs show attachment behaviour towards their caregivers, which shows parallels with the attachment between infants and their parents [84,85,86]. The presence of the owner can reduce the extent of the stress response in new situations (“secure base effect”, [84]) or when the dog is frightened and seeks out the owner (“safe haven effect”, [87]). One study compared physiological parameters in dogs that were either kennelled at a clinic for 12 h or were brought in immediately prior to gonadectomy. Dogs that spent the night in the clinic showed a significantly higher oxidative stress index and increased cortisol levels in a blood sample taken just before surgery than those that were brought in directly before surgery [88]. In another study, dogs had higher blood pressure and higher heart rate in the absence of their owners compared to when the owners were present [89]. Consequently, whenever possible, enabling the owner to be present is an important factor in reducing stress in dogs [7].

Csoltova et al. [90] compared signs of stress in dogs during a standardised clinical examination, during which owners were either present but passive (3 m away from the table) or petted and talked to their dog while on the table. In the ‘contact’ condition, dogs made significantly fewer attempts to jump off the table and exhibited a significantly lower heart rate and a significantly lower eye temperature, measured by a thermographic camera, than in the ‘non-contact’ condition, indicating a lower stress level when having physical contact with the owner [90].

One exception to owners’ beneficial influence is when the owner is very stressed or anxious him- or herself. In unclear situations, dogs will look back at their caregiver to better assess the situation and adapt their behaviour according to the owner’s emotional expressions [91,92]. If the owner signals that the situation is dangerous or scary, this may indeed affect the dog negatively, and in rare cases, treatment in the absence of the owner may be preferable [7].

Furthermore, some patients may show less aggression when the owner is absent [36]. This does not necessarily mean that they are less fearful, but they adopt a different coping strategy [24]. While treating the pet in the owner’s absence in these cases may seem a solution, it carries the risk of traumatising the animal. Moreover, if the animal learns that freezing is not successful in avoiding the perceived threat, it may opt for a different strategy next time, so that handling may become progressively more difficult [24]. Thus, re-planning the visit with premedication or behaviour modification, as described in Section 9.1, or the use of chemical restraint if the procedure cannot be postponed is most likely more conducive to the animal’s welfare [24,49] and does not carry the risk of the owner assuming that the pet is being mishandled when they hear crying, barking or growling next door [36].

Studies on the influence of the owner on the behaviour of cats in the veterinary situation are still lacking. However, previous study results suggest that cats also form a bond with their owners [93,94] and that the owner is important to the cat, although they generally seem to be affected less by separation than dogs [95].

## 8. Inpatients

Hospitalisation and the separation from the owner are highly stressful for many animals [88]. Stress can have a negative effect on wound healing, cardiovascular health and the gastrointestinal system. In the long term, immunosuppression is also possible (reviewed by [96]). Stress reducing measures are therefore especially important for hospitalised patients.

Similar to the waiting room, dogs and cats should be housed separately, and dog barks should ideally not be audible in cat holding areas [57]. For both species, it is recommended that the cages or kennels face the wall, so that there is no visual contact with other animals. Predictability of daily routines and control of stressors (e.g., minimising noise during cleaning activities) are associated with improved welfare, and the animals are more likely to make contact with the caregivers [57].

A major problem when housing dogs is the noise exposure and restlessness caused by barking and other forms of vocalisation. Studies from shelter settings indicate that playing classical music [97] or audio books [98] and offering activity feeding [99] can reduce vocalisations. Feeding enrichment, such as by the provision of puzzle toys filled with food, is furthermore valuable by reducing boredom [40]. Regular positive contact with caregivers, e.g., stroking, walking, presence of a human, are associated with reduced stress levels in kennelled dogs and cats [100,101,102].

Cats have an instinctive need to hide in threatening situations; thus, the availability of hiding places such as by providing boxes, or simply by covering (half of) the cage with a towel is important for their welfare [40]. Studies demonstrated that hospitalised cats with a hiding box in the cage or a towel draped over the cage had lower heart rates compared to controls without hiding places. In addition, cats that had a box available showed fewer behavioural stress signs than the control group [103,104]. A trend in the same direction was observed in the group with the towel [104]. By using towels to cover only half the cage, visual access for the clinic staff is possible, while still giving the cat a sense of security [105].

Especially for inpatients, familiar odours (e.g., blankets, toys) are recommended to provide security [48]. If there are concerns that personal belongings may be lost or have to be disposed of for hygienic reasons, owners could be asked to bring old towels with their scent. These can be cut into smaller strips and stored in closed containers to best retain the smell. The used strips can be replaced as necessary so the animal still benefits from the owner’s scent [7].

When an animal has a known tendency to react aggressively, putting a sign on its cage and recording this in its medical history helps to ensure that all staff members are aware of the animal’s special needs. Leaning into the kennel or blocking the doorway should be avoided as it may cause the animal to feel trapped, increasing the risk of a bite [61]. Talking softly while adopting a sideways rather than frontal stance may encourage the animal to approach; this is much preferable to reaching into the cage to grab the animal [36]. With fearful or aggressive dogs, it may also be helpful to leave a lightweight lead attached to the collar or preferably harness to facilitate handling [61].

## 9. Prevention and Training Measures

Whenever an animal comes to the practice, every effort should be made to keep it feeling safe and to create positive experiences. A simple and time-efficient way is to feed the animals during the examination [36,62]. The first impression is particularly important—thus, the first examination or vaccination can have a lasting effect on a dog’s behaviour [3]. As described above, painful experiences during vaccination can be minimised by tactile distraction as well as feeding the animal through the procedure [7].

**Figure 8:**
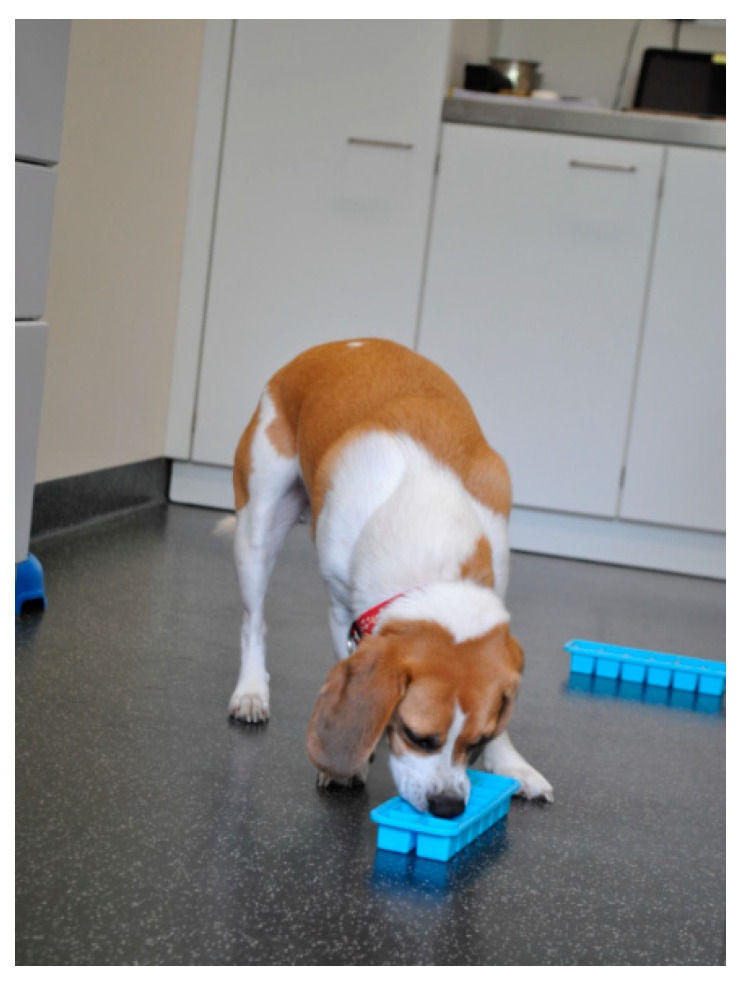
‘Happy Visits’ enable positive experiences in the clinic environment. In this setup, several forms filled with lickable food (normal wet food works well) are spread throughout the examination room and the dog is free to choose without any pressure. A reduced version of such a ‘happy visit’ can also be offered during normal consultations—the dog can lick some food while the veterinarian is taking the history. Photo: Stefanie Riemer.

Veterinary personnel should be trained in desensitisation/counterconditioning techniques and use it whenever possible (c.f. [60]). Additionally, ‘happy visits’ can be used to create a more positive emotional state in the clinic (Figure 8). Owners can be encouraged to bring their animals to the clinic just to greet the reception staff, be rewarded for stepping on the scale, or briefly visit an examination room where a staff member may greet them and give them treats [36]. Such visits to the practice without any examinations or treatments reduce the risk that the animal will later show conditioned fears (e.g., in connection with injections) [3], enable positive experiences and can also be incorporated into a training plan for already fearful dogs [36].

In addition, owners play an important role in preparing their pets for veterinary visits. Ideally, puppies and kittens would be trained to accept touching of the whole body and examinations, such as opening the mouth and handling the paws from a young age [3]. This is best done according to the principle of desensitisation/counterconditioning (see Section 9.1, c.f. [60]).

Carrier training should be essential for any cat (and dog) [49,51]. To this end, owners should create positive associations with the carrier at home, such as by placing it in the living room and regularly offering incentives such as food, toys or petting in and around the carrier [24,28,37,52]. Voluntary entry and remaining in the closed carrier can furthermore be trained using positive reinforcement [52].

The choice of a carrier model with an easily and quietly removable top and an additional opening at the top facilitates removing and returning the cat from/to the carrier [39,51]. Travel sickness can be prevented by medication such as Maropitant (approved for dogs; also recommended for cats) [29]. Each dog should also receive muzzle training (Figure 7) to ensure that the muzzle does not act as an additional stressor should it become necessary [60].

During the veterinary visit itself, owners should convey positive emotions and confidence, which can be aided by a sympathetic stance from the veterinary team and by advising owners on how to help to make the visit most pleasant for their animal. This includes bringing favoured food, chews, toys and a familiar blanket [7,29,36,62], as well as refraining from feeding a full meal prior to the visit [29,36], so that counterconditioning methods using food treats can be implemented while the animal has some appetite [106].

### 9.1. Behaviour Modification Techniques

The primary recommended behavioural techniques to reduce fear responses in animals are counterconditioning and desensitisation (see below). Mere habituation (reactions to a stimulus decline when it is presented repeatedly or over a longer period of time [107] is usually not sufficient to improve fear at the veterinarian’s (e.g., [90]). Conversely, sensitisation may occur and the individual’s response to the stimulus may become stronger [107]. Flooding (the animal is exposed to a fear-inducing stimulus at full intensity and is prevented from escaping, with the aim of extinguishing the fear response) creates a situation of extreme fear, can lead to panic reactions and carries the risk of aggravating the fear. Flooding often inadvertently happens during veterinary examinations or treatments, but it is no longer a recommended technique in veterinary behavioural therapy due to animal welfare reasons [106].

With desensitisation, the animal is exposed to a stimulus at such a low intensity that it does not induce any fear. Stimulus intensity is then gradually increased as long as the animal remains relaxed. The aim is to extinguish the initial fear response over the course of numerous repetitions. Critical to the success of desensitisation is that the stimulus must be presented at a level at which the animal remains relaxed. If stimulus intensity is too high, this can inadvertently lead to sensitisation. As a consequence, the individual’s reaction to the stimulus increases [107]. Desensitisation is most effective when combined with counterconditioning [106].

In counterconditioning, the fear-eliciting stimulus is paired with desirable consequences such as food or play, in order to replace the association with a positive one [108]. The (former) trigger should be reliably followed by a reward each time in order to build a strong positive association. Counterconditioning works best if carried out in parallel with desensitisation, i.e., stimulus intensity should initially be kept low so as not to elicit a fear response and can slowly be increased over the course of training (see [106]).

**Figure 9:**
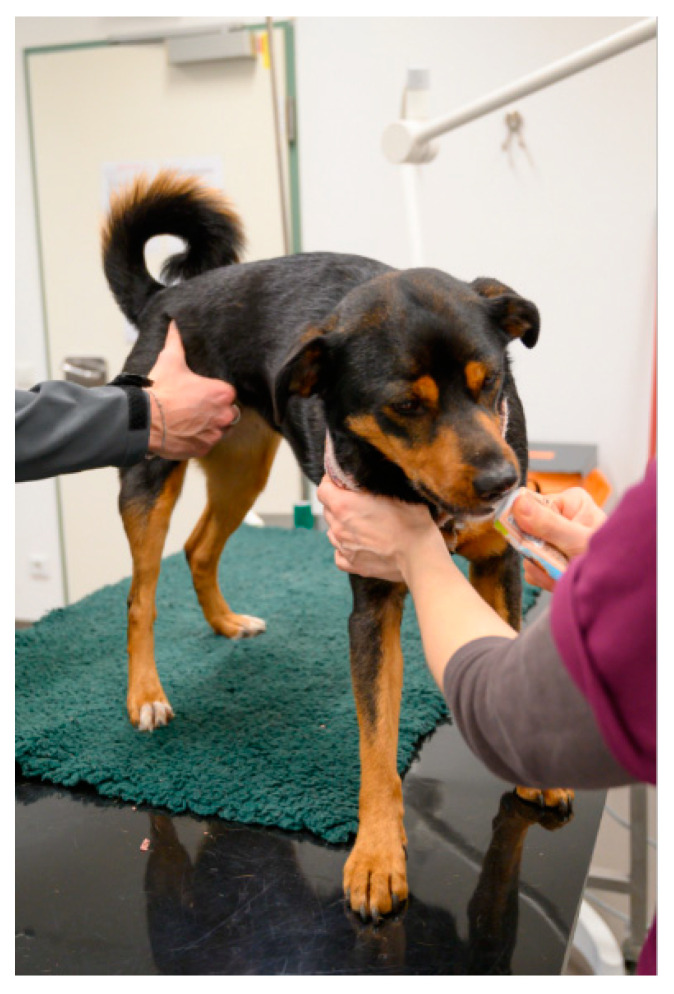
During counterconditioning, a potentially unpleasant or fear-inducing stimulus is paired with a high-value incentive. For optimal results, the start of the potentially aversive stimulus should precede the start of the positive desired stimulus, so that it becomes a predictor of something positive. Thus, the ideal sequence of events is as follows: start touch, then start feeding and continue feeding throughout the touch. When you stop touching, also stop feeding. Photo: Christine Arhant, Vetmeduni Vienna.

To achieve this in a veterinary context, the examination and treatment are broken down into small approximations, and each step is immediately followed by a reward, usually food (Figure 9). The approximations are designed so that the animal shows no or hardly any signs of stress, e.g., the examiner might start by moving the hand towards the animal (without touching) followed by a reward and then touching a non-sensitive area followed by a reward. Each further step, such as moving the hand to the target area and gradually increasing time and intensity of the examination, should be followed by a reward. As each step is repeated several times, the animal should become more relaxed—an increase in body tension can be an indication of sensitisation taking place. In the latter case, even smaller steps have to be taken, i.e., the units should be made even shorter or less intense, or other strategies (for example, additional administration of anxiolytic medication) should be used [27,37,38]. Detailed protocols for/counterconditioning in a veterinary context are given in [60].

While relaxation should always be the goal, in classical counterconditioning, a reward is always given contingent on the stimulus, regardless of the animal’s behaviour (see [106]). In the past, this method was used less, because it was wrongly assumed that giving rewards when the animal showed fearful behaviour could reinforce the fear. However, a negative emotion cannot be rendered more negative by adding something positive; on the contrary, the positive experiences can improve the emotional state [106]. Thus, in classical counterconditioning, one works first on the level of emotions, which subsequently leads to a behavioural change [106]. Of course, this does not mean that the training should be conducted at a level that is over the individual animal’s threshold, but when it does happen, a reward should be given anyway, as the best success can be achieved when the contingency between the fear-inducing stimulus and the reward is reliable [106]. If the animal does react fearfully or even aggressively, this is a clear sign that the situation needs to be adjusted in a way that helps the animal to keep feeling safe [109], such as by changing the training plan or the use of anxiolytics when necessary [27,37,38].

Crucial to the success of counterconditioning is that the reward used is of extremely high value to the animal to produce a strong conditioned positive response [106]. Highly palatable food is particularly suitable, but also play and petting can be used [60]. Counterconditioning is most effective when the potentially aversive stimulus, such as for example, touch of the animal by a veterinarian, is a predictor of something positive. Thus the order should be touch, then feed, and not the other way around [60].

Note that his method should not only be applied in the treatment of already existing fears but can be used in the same way to prevent negative associations [60]; also see [110] on the benefit of preventative counterconditioning.

### 9.2. Preventing a Resurgence of Fear

From a neurobiological point of view, fear extinction is only successful if a so-called extinction memory is formed and can be retrieved, which ‘overlays’ the fear memory. However, negative memories are never completely forgotten, since different regions of the brain are responsible for fear and its inhibition [111]. Under acute stress, when the animal has not been exposed to the stimulus for a prolonged time, and when the trigger occurs unexpectedly or in a new context, a resurgence of fear commonly occurs [111,112,113]. A very important factor in fear extinction is therefore generalisation (e.g., conducting an examination in different places and by different people, etc.)—this should, however, be done very carefully to prevent a new fear reaction [114].

Some patients are so fearful that the only realistic goal, both during the visit to the veterinarian and during training at home, is to teach them to tolerate the measures necessary for intramuscular sedation [60]. Under certain circumstances for some individuals, a kind of “emergency” management may be best, such as injecting a sedative as quickly as possible outside the surgery, in a very distracting environment, without prior palpation of the muscle [60]; however, owners should be informed that the risk is increased if sedation is administered without prior examination [115].

### 9.3. Cooperative Care Training

**Figure 10:**
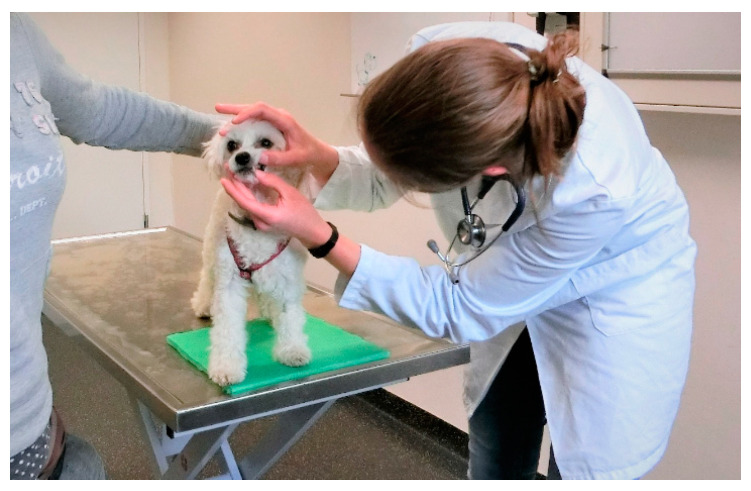
Animals can be trained to perform a ‘consent behaviour’ such as stepping onto a target mat to indicate when they are ready for manipulations. When the animal interrupts the behaviour, any manipulations are stopped, thus giving the animal control. Photo: Stefanie Riemer.

Cooperative care training has been pioneered by zoos and other institutions that keep wild animals, enabling medical procedures, including invasive ones such as blood sampling, without the necessity of sedation (e.g., [116]). Beyond desensitisation and classical counterconditioning, animals can be trained to give their consent to an action by showing an operantly trained behaviour (for example, stepping onto a target mat or placing the chin on a hand or target) (Figure 10). At the same time, the animals can also stop the procedure at any time by discontinuing the learned operant behaviour. This training is always combined with a desensitisation/counterconditioning approach, where the animals learn to tolerate handling and medical procedures in small steps [60]. However, if a procedure has to be carried out to which the animal will likely not consent (all the way through to the end) and breaking off is not an option, the target should not be used. Instead, training animals with positive reinforcement to accept different types of restraint should also be part of preparing animals for veterinary visits, so that it is less stressful when needed [114].

Further ways to facilitate medical and husbandry procedures through cooperative care training include the training of behaviours such as opening the mouth, presenting a particular body part, ingesting medication, accepting eye and ear drops or shortening the nails by scratching on a scratchboard (a board with sandpaper) [60,117].

A study on laboratory cats demonstrated that training kittens to accept venipuncture using a gradual approach was associated with less stress during blood sampling when the cats were adults [118]. While no studies on cooperative care training in adult pet cats are available, one study has demonstrated that using positive reinforcement to train cats to voluntarily enter and stay in a cat carrier and habituate them to the car (28 sessions within six weeks) was associated with reduced stress during transport and less time required for the clinical examination, suggesting lower stress and thus increased willingness to cooperate [52].

If cooperative care training is carried out by the owners themselves, good guidance from the veterinary team or dog trainers/behaviourists, as well as high owner compliance, is essential. In a study in which owners were instructed to train their dogs to accept various examination steps over a period of four weeks, using written and video instructions, only minor positive effects were found, despite additional ‘happy visits’ to the clinic [119]. Owners should therefore be prepared for the necessity of longer-term training, ideally in combination with enabling positive experiences including members of the veterinary team.

Often, cooperative care training is recommended as part of a behaviour modification programme when animals are already fearful at the veterinary practice. For successful implementation in the veterinary setting, the cooperation of the practice team is thus essential to enable a successful transfer of the training from the home to the veterinary environment. Training at the veterinary practice/with the veterinary team can be seen as a measure to promote client loyalty or can be charged as a veterinary behavioural/training consultation, depending on the effort involved [114].

## 10. Medication

With some animals, management and stress-reducing techniques are not sufficient to reduce fear and anxiety. If patients cannot be examined and/or treated effectively and safely, medication should be considered. To achieve the best effect, anxiolytic medication should ideally be given before the animal becomes overly aroused and stressed [24]. Therefore, pre-visit anxiolytic medication administered by the owners is typically most effective [38].

It is important to emphasise that the use of psychotropic medication must never replace responsible and welfare-compliant handling and interactions with the animal. Rather, it is a supportive measure with the aim of reducing stress and fear without impairing normal behaviour [27] and to reduce the suffering of the animal, improve its welfare and facilitate behavioural training [60]. Hence, behavioural modification techniques alongside medication play an important part of the treatment plan. Through the concomitant use of anxiolytic medication with behavioural training, learning can be facilitated [120].

### 10.1. Application Options

When choosing an appropriate pre-visit medication, the purpose of the veterinary visit and owners’ ability to administer different types of medications need to be considered. For preventive checkups or vaccinations, the animal generally does not need to be fasted and medication can easily be administered with food. If an animal has to remain fasted, transmucosal forms of application (e.g., in gel form) or medication that is soluble in a small amount of water would be preferable. While gels have to be applied to the animal’s gums by the owner, aqueous solutions may not be palatable for animals (e.g., bitter taste of gabapentin). The (forced) oral application of aqueous solutions is difficult for many owners and can lead to significant stress both for the owner and the animal. Therefore a pre-visit discussion with the owner considering the reason for the visit and the application skills of the owner, including the tolerance of the animal to manipulations around its head, is recommended to choose an appropriate medication [114].

In the following section, the most relevant classes of anxiolytic medications and the scientific evidence for their effectiveness in a veterinary setting are reviewed. The use of polypharmacy, detailed dosage recommendations, potential side effects, interactions and contraindications in animals with impaired kidney, liver or heart function are not discussed in this review; a consultation with a veterinary behaviour specialist or a pharmacology textbook is advised [114].

### 10.2. Selected Medication Options

#### 10.2.1. Trazodone

Trazodone belongs to the class of serotonin antagonists and serotonin reuptake inhibitors. High dosages have antidepressant effects, while low and medium doses are used in the context of veterinary fear and other situational fear states [121,122]. In hospitalised dogs, an application of Trazodone led to a reduction of various stress signs, including flattened ears, trembling, panting, licking lips, averting the gaze, pacing, growling and snapping, after 90 min [123]. In cats, a single administration of Trazodone 1–1.5 h before transport to the veterinary practice resulted in a reduction of fear indicators during travel and increased compliance during the physical examination [124].

#### 10.2.2. Alpha 2 Adrenoreceptor Agonists

Dexmedetomidine transmucosal gel has been approved as Sileo© for noise fears in dogs [125]. In the veterinary context, the use of Sileo© as pre-visit medication increased dogs’ compliance and reduced heart rate by up to 30 bpm during the examination compared to a baseline examination [126]. A recent crossover, double-blinded, placebo-controlled study demonstrated that the administration of dexmedetomidine oromucosal gel was associated with reduced vocalisations (such as whining, yelping, and grumbling), avoidance behaviours and panting, trembling, urination and defecation during a veterinary examination compared to placebo [127].

Dexmedetomidine could also reduce car travel-related problems. In a triple-blinded crossover study, dogs showed a significant reduction in panting and yawning when administered dexmedetomidine one hour before the start of a 10-min car ride [128].

In cats, there are numerous studies on the good sedative and analgesic effect of intravenous or intramuscular use of dexmedetomidine (e.g., during anaesthesia) [129,130,131,132], but the high probability of inducing vomiting (e.g., [133,134]) is considered counterproductive for a stress-reducing function in the awake animal [114].

Another alpha 2 adrenoreceptor agonist is clonidine. Despite its use in veterinary behaviour medicine, only one open label study of the efficacy of clonidine in dogs diagnosed with different forms of anxiety has been published [135]. No details about its effect during veterinary visits are available so far. Given this lack of studies in dogs and cats, the use of dexmedetomidine might be preferable for dogs. However, clonidine tablets may be advantageous if a gel application by the owner is not possible (e.g., aggressive behaviour of the dog when its mouth is manipulated) [114].

#### 10.2.3. Gabapentin

The anxiolytic effects of the anticonvulsant and structural GABA analogue gabapentin are mainly known from humans [136] and cats [137]. In cats, a single administration of gabapentin 90 min before transport to the veterinary practice was associated with significantly lower stress scores (assessed by the owners) and compliance scores (assessed by the vets) [137]. Gabapentin powder from the opened capsule can be administered with tasty liquid, such as tuna juice or with wet food (note that gabapentin powder is bitter and can lead to increased salivation) [114]. In addition, a first dose can be administered on the evening before the veterinary visit [138]. Owners must be informed that there is an increased risk of falling from elevated areas and that cats should remain under supervision after administration to prevent climbing and potential falls [138]. For dogs, studies on the anxiolytic effects of gabapentin are still lacking. In the experience of the authors, dosages at the higher end of the range may be necessary to achieve an anxiolytic effect [114].

#### 10.2.4. Benzodiazepines

Benzodiazepines are anxiolytic drugs with a rapid onset of action that are suitable for short- to mid-term use. The most important benzodiazepines are alprazolam, lorazepam and diazepam (the latter is suitable only for dogs, as it can cause hepatic necrosis in cats). As no published studies are available on the effect of benzodiazepines on fear in dogs and cats in a veterinary context, and because clinical trial data are generally scarce, recommendations are mainly based on the experience of experts (e.g., [27,36,123]). Alprazolam is recommended for panic-like conditions [139] and was reported to be highly effective for noise fears [140]. However, the owners should be made aware that paradoxical reactions, such as increased arousal, restlessness, and insomnia, may occur [36,122]. Moreover, in animals prone to showing fear-based aggression, benzodiazepines might heighten aggressive reactions due to disinhibition [36]. Administering a test dose to assess any undesirable side effects before an imminent visit to the veterinarian is particularly important for this class of drugs [122].

#### 10.2.5. Why Is Acepromazine Not State of the Art Anymore?

Acepromazine belongs to the class of phenothiazines. It causes sedation, muscle relaxation and a reduction of spontaneous activity. No anxiolytic effect has been demonstrated to date; therefore, acepromazine should be rejected as a single medication for the treatment of any form of fear related problems [29]. Acepromazine was not associated with a reduction of stress parameters compared to placebo when used during a flight in dogs [141]. In addition, bitches treated with acepromazine prior to ovariohysterectomy had significantly higher blood concentrations of epinephrine, norepinephrine and cortisol when compared to a medetomidine group, indicating higher stress levels [142]. The administration of acepromazine may even lead to an aggravation of fear symptoms [27]. So, it is not surprising that “high psychological arousal” is explicitly listed under counter indication in the instruction leaflet of the licensed product (Sedalin, Vetoquinol, France) for the European Union.

## 11. Pheromone Therapy

Synthetic pheromone products are often suggested as adjunctive intervention. They are available as sprays and diffusers for dogs and cats and as a collar for dogs. Synthetic analogues for dogs correspond to the “Dog Appeasing Pheromone” (DAP) from the mammary glands of the lactating bitch (Adaptil^®^) and for cats to feline facial pheromone F3 (Feliway^®^) and are assumed to have a calming effect on the respective species [26]. While some studies indicated beneficial effects of Adaptil^®^ and Feliway^®^ on stress signs (but not aggression) in dogs and cats at the veterinarian [143,144,145], another study found no positive effect of Feliway^®^ on stress parameters in cats [146]. One study indicated a reduction in pacing, elimination and excessive licking in hospitalised dogs under DAP compared to the placebo group [147]. Another assessed whether DAP influenced behavioural and physiological changes in dogs after neutering compared to behaviour before the operation. No effects of pheromone treatment on serum cortisol or serum glucose were found; however, the prolactin concentration decreased less in the DAP group than in the placebo group [148]. Out of a large number of measured variables, the only behavioural differences noted were in visual exploration behaviour and alertness, with less decreases in the DAP group [148]. 

A critical review of studies on the use of pheromones specifically for hospitalised veterinary patients concluded that the evidence so far is insufficient to demonstrate the effectiveness of pheromone products in reducing distress in dogs and cats. This conclusion was not only based on a critique of study design and reporting of the analysis of the included studies (e.g., measuring a large number of variables without correcting for type 1 error), but also because for Feliway spray, the manufacturer stated that strong-smelling disinfectants, bleach, biological washing powder, detergents or deodorisers—products that are commonly used in a veterinary setting—can interfere with the product’s functioning (reviewed in [105]). Moreover, several recent controlled studies could not confirm calming effects of Adaptil^®^ on dogs in other contexts including separation from the owner and shelter housing [149,150,151]. According to a systematic review, the effectiveness of pheromones in dogs and cats on fear behaviour could not be demonstrated in the majority of studies due to a lack of adherence to scientific quality criteria [152].

## 12. Conclusions

Veterinarians are increasingly required to consider not only the physical, but also the psychological health of their patients. Creating low-stress veterinary visits has immediate as well as long-term benefits on the patients’ welfare and health, as well as reducing risk of injury to the staff. Considerations range from structural adaptations (e.g., set-up of the waiting area and inpatient wards) to using low-stress handling approaches, recognising signs of stress, and adjusting the procedure accordingly. However, not only should stressful interactions be avoided, but pleasant emotions and emotional resilience can be promoted by using incentives (high-value food, toys or petting) to create positive associations throughout the animal’s visit. Desensitisation/ counterconditioning and enabling ‘happy visits’ are recommended both for the prevention and the treatment of fears associated with veterinary visits. When management and low-stress handling methods are not sufficient to moderate an animal’s fear, anxiolytic medication is indicated alongside a behavioural treatment plan. Although accommodating perceived “difficult” animals may initially cost more time, investing in individual routines and enabling the animals to feel safe lead to more efficient examinations and treatment in the long term [3,60] and reduces the risk of injury to the staff [29,39,54]. Relationship building measures not only increase client loyalty [38], but also improve staff satisfaction (reviewed in [40]). Thus, a good relationship between the veterinary team, owners and their pets leads to a better lifelong health care for veterinary patients and is of benefit to everyone involved [7,114].

## 13. Further information

The following books go into more practical detail on how to implement some of the discussed measures: (1) Howell, A.; Feyrecilde, M. Cooperative veterinary care; Wiley Blackwell, 2018; (2) Yin, S. Low stress handling, restraint and behavior modification of cats & dogs; Cattle Dog Publishing: Davis, 2009.

Furthermore, several online training programmes are available on this topic: (1) FearFree: https://fearfreepets.com; (2) Cat Friendly Clinic: https://catfriendlyclinic.org; (3) Better Vet Visits: https://karenpryoracademy.com/courses/better-vet/; (4) Low Stress Handling^®^ University: https://lowstresshandling.com.

## Data Availability

Data sharing is not applicable to this article. No new data were created or analysed for this manuscript.
